# An Efficient and Effective Image Decolorization Algorithm Based on Cumulative Distribution Function

**DOI:** 10.3390/jimaging10030051

**Published:** 2024-02-20

**Authors:** Tirui Wu, Ciaran Eising, Martin Glavin, Edward Jones

**Affiliations:** 1 School of Engineering, University of Galway, H91 TK33 Galway, Ireland; martin.glavin@universityofgalway.ie (M.G.); edward.jones@universityofgalway.ie (E.J.); 2 Department of Electronic and Computer Engineering, University of Limerick, V94 T9PX Limerick, Ireland; ciaran.eising@ul.ie

**Keywords:** cumulative distribution function, edge recall ratio, gradient recall ratio, image contrast preservation, image decolorization

## Abstract

Image decolorization is an image pre-processing step which is widely used in image analysis, computer vision, and printing applications. The most commonly used methods give each color channel (e.g., the R component in RGB format, or the Y component of an image in CIE-XYZ format) a constant weight without considering image content. This approach is simple and fast, but it may cause significant information loss when images contain too many isoluminant colors. In this paper, we propose a new method which is not only efficient, but also can preserve a higher level of image contrast and detail than the traditional methods. It uses the information from the cumulative distribution function (CDF) of the information in each color channel to compute a weight for each pixel in each color channel. Then, these weights are used to combine the three color channels (red, green, and blue) to obtain the final grayscale value. The algorithm works in RGB color space directly without any color conversion. In order to evaluate the proposed algorithm objectively, two new metrics are also developed. Experimental results show that the proposed algorithm can run as efficiently as the traditional methods and obtain the best overall performance across four different metrics.

## 1. Introduction

**Figure 1:**
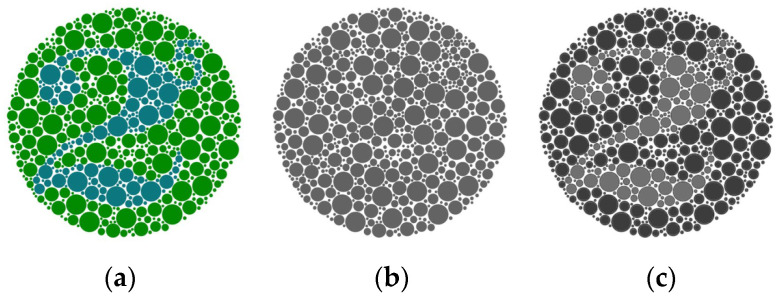
Image decolorization. (**a**) The input image, (**b**) grayscale image generated according to [20], (**c**) proposed method.

Despite color images dominating our daily life nowadays, grayscale images are still widely used in image analysis and computer vision applications, hence the need for image decolorization (ID) algorithms. Many important computer vision algorithms are designed for grayscale images, such as Haar Cascade Classifier for face detection [1], Histogram-of-Gradients (HoG) [2], Scale-Invariant Feature Transform (SIFT) [3], Local Binary Pattern (LBP) [4], edge detection [5,6], line detection [7], corner detection [8,9], and keypoint descriptors [10,11,12]. These algorithms are still wildly used in many visual applications, such as visual Simultaneous Localization and Mapping (vSLAM) [13,14,15,16,17] and Structure from Motion (SfM) [18,19]. They heavily rely on image details (e.g., edges and corners) and image contrast to achieve good performance. Also, ID is a critical step for printing applications when grayscale printing is needed. Traditional methods, for example extracting the Y component of an image in CIE-XYZ color space [20], which give each color channel a fixed coefficient to compute the weighted average of the three color channels, produce acceptable results most of the time. But sometimes, such methods may produce unacceptable output, as the example in Figure 1 below shows, where a lot of image details are lost in the image in Figure 1b.

In this paper, we present an efficient and effective ID algorithm. It can retain more image contrast and details than the traditional method from the original color image, as Figure 1c shows. It uses the information from the cumulative distribution function (CDF) of each channel image to compute a weight for each pixel in each color channel. Then, these weights are used to combine the three color channels (red, green, and blue) to obtain the final grayscale value. Because of its simplicity, the computational cost is nearly as low as that of the traditional methods, which makes it suitable for practical applications on computationally constrained platforms, such as mobile phones and edge computing devices. In order to measure its performance objectively, two new metrics are also proposed.

The contributions of this work are summarized as follows:

- We propose a fast, efficient, and effective ID algorithm to address the computational limitations of existing ID algorithms that limit their practical usage. The proposed algorithm has linear time complexity and can achieve comparable runtime performance to traditional methods;
- We propose two new objective metrics to measure the performance of our ID algorithm. By combining these new metrics with existing metrics, a more comprehensive evaluation of ID algorithm performance may be obtained.

The rest of this paper is organized as follows. Section 2 briefly reviews related work. Section 3 describes the proposed method. Section 4 describes the two proposed objective metrics and demonstrates the effectiveness and efficiency of the proposed method, and Section 5 provides the conclusions. 

## 2. Related Work

Several techniques have been proposed to retain more information from the original color image. They roughly are categorized into two groups: approaches based on the use of local information in the image [21,22,23,24,25,26,27,28] and based on global information [29,30,31,32,33,34,35,36]. The local approaches enhance the final grayscale output with local information, like chrominance edges and high-frequency image details, while global approaches combine both local and non-local contrast, structure, and illumination for color compression [35].

Among the first group, Bala and Eschach [21] proposed a method to preserve the local distinction between adjacent colors by integrating high-frequency chrominance information into the luminance channel. The problem with this method is that it may introduce lots of undesired artifacts around image edges due to the high frequency filter used in the algorithm. In [22], Gooch et al. take the conversion process as an optimization problem: the gray value of each pixel is iteratively adjusted to minimize an objective function, which is based on the local contrasts between all the pixel pairs. However, the high computational complexity of this method makes it impractical for high resolution images. Another optimization-based method was proposed by Rasche et al. [23] to minimize the error function based on matching the gray differences to the corresponding color differences to find an optimal conversion. The method can preserve image contrast and maintain luminance consistency, but its computational cost is high, which limits its use. Queiroz and Braun [26] developed a reversible method which is based on mapping colors to low-visibility high-frequency textures that are applied onto the gray image to achieve image decolorization. The Wavelet transform is used in the conversion process, which adds computational cost. Wu and Toet [27] proposed a method based on multiresolution channel fusion for ID. This method computes weight maps to fuse the three color channels based on several well-defined metrics. Another fusion-based algorithm was recently proposed in [28]. Such fusion-based methods are effective, but not efficient; they require a lot of computational resources for image decomposition and reconstruction when the input image is large. One recent local approach in [37] uses boundary points as optimization targets so that the output grayscale image can maximally preserve the boundary points of the color image.

As for the global approaches, Grundland and Dodgson [29] introduced a fast method to decolorize color images. It combines novel techniques for image sampling and dimensionality reduction to achieve efficient conversion. In addition to its speed and simplicity, the algorithm has the advantages of continuous mapping, global consistency, and grayscale preservation, as well as predictable luminance, saturation, and hue ordering properties. In [32], a global–local ID method was proposed. The method first globally assigns gray values and determines color ordering, then secondly locally enhances the grayscale to reproduce the original contrast. In [33,34], a convolutional neural network is used to extract high-level abstract features from the input color image to achieve effective contrast preservation. In [35], an invertible image decolorization is proposed to produce invertible grayscale images using invertible neural networks (INNs). Ref. [36] uses deep neural networks to extract content information based on the human vision system to select suitable grayscales for decolorization. The big disadvantage with this approach is that it needs to run the optimization process with each single image, which is a time-consuming process even with high performance GPU. Recently, a two-stage algorithm based on the image histogram and local variance maximization to preserve more image information was proposed in [38]. While this approach provides good performance, the use of optimization results in a relatively high computational cost and limits its practical application. 

Most of the algorithms described above carry a higher computational cost than traditional methods. Therefore, it is challenging to use them in many real-time applications. While, on the one hand, modern computing devices with embedded cameras, e.g., smartphones or other edge devices, have substantial computing resources, on the other hand, there is an increasing use of high-resolution cameras, which results in a substantial pre-processing load. This is one reason why traditional algorithms are still widely used in many different applications. Our aim in this work is to propose an algorithm which can run nearly as fast as the traditional methods, preserve more image contrast and image details than the traditional methods, resulting in higher functional performance, and be implemented easily.

## 3. Proposed Algorithm

**Figure 2:**
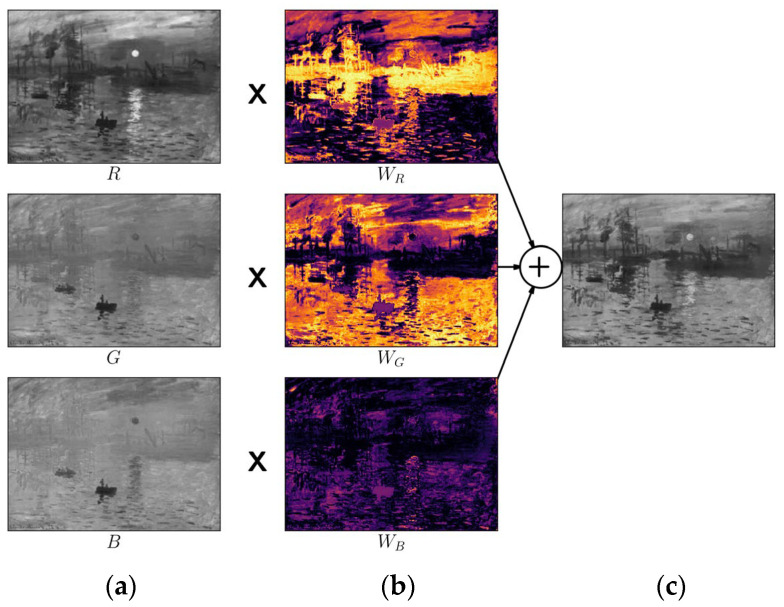
Output of each stage of the proposed method. (**a**) Generate the individual channel images (R, G, B); (**b**) calculate weights for each channel image with Equations (2) and (3); (**c**) combine weighted channels to produce output grayscale image.

In this section, we will discuss the proposed method in detail. Figure 2 summarizes the workflow of the proposed method. In the first step, we split the input image into three separate channel images: red, green, and blue. Then, we derive weight maps based on the CDF in each channel. In the final step, we compute the weighted sum of the individual channels to obtain the final grayscale output. 

### 3.1. Algorithm Description

**Figure 3:**
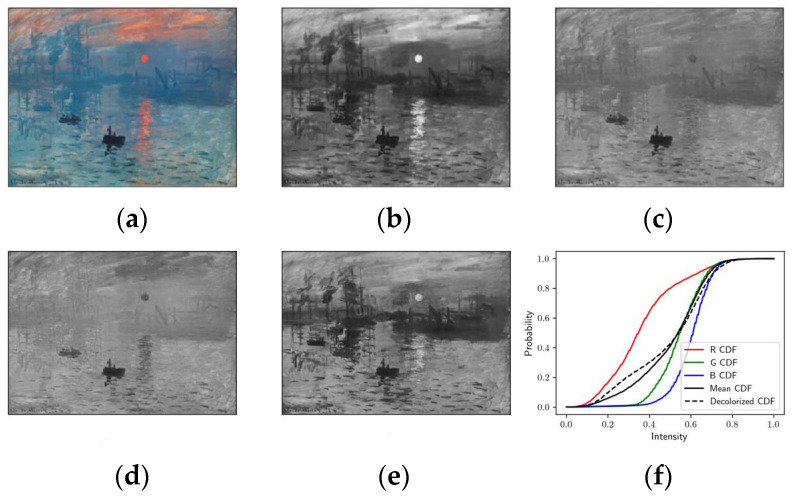
Cumulative distribution function of each channel for a sample image. (**a**) is the input image; (**b**–**d**) are the intensities of the red, green, and blue channels, respectively; (**e**) the final decolorized image; (**f**) the corresponding CDFs for the three channels. Mean CDF is the average of the three individual channel CDFs. The decolorized CDF is also shown, and not only approximates the mean CDF but also captures some of the details of the three color channel CDFs.

For a color image, each color channel (also referred to as a channel image) will have a different probability mass function (PMF, also called a histogram in image processing) which results in different cumulative distribution function (CDF), as shown in the example in Figure 3. The CDF can be computed by running a cumulative sum on the PMF ($CDF\left[i\right]=\sum_{k=0}^{i}PMF[k]$, where $i\in \left[0,L-1\right]$, and *L* is the number of bins in the PMF). From the CDF curves for each color channel (shown in Figure 3f), we can obtain some information about each color channel image. For example, red channel images are dark because more than half of the pixel intensities are less than 100 and green/blue channel images are light because more than half of the pixels have an intensity larger than 150 (Figure 3b–d). Also, the red channel has higher image contrast than the green/blue channels, reflected in its CDF curve having a shallower slope. So, the CDF contains some useful information about image content.

Inspired by histogram equalization (HE), which uses CDF as a mapping function to redistribute pixel intensities to increase image contrast, we design a simple strategy to compute a weight map for each channel image by calculating an optimal CDF which can approximate the three channel image CDFs and then computing a weight for each pixel color component based on differences between the optimal CDF and the channel image CDFs. We build an optimal CDF by averaging the three channel CDFs so that each element in this optimal CDF has minimum total $L_{2}$ distance to the corresponding elements in the three channel CDFs. This can be obtained by solving a simple minimization problem: ${argmin}_{{CDF}_{opt}}\sum_{c\in \{r,g,b\}}{\left({CDF}_{opt}-{CDF}_{c}\right)}^{2}$. The solution is:

(1) $${CDF}_{opt}\left[i\right]=\frac{\sum_{c\in \{r,g,b\}}{CDF}_{c}\left[i\right]}{3}$$

where i∈[0, L−1] is the pixel intensity, *L* is the maximum level of pixel intensity (e.g., for 8-bit depth image, $L=2^{8}$), and ${CDF}_{c}$ is the CDF of the individual channel image. After we have an optimal CDF, we can compute the weight for each level in each color channel with the following equations:

(2) $$W_{c}\left[i\right]=e^{-\left|{CDF}_{c}[i]-{CDF}_{opt}[i]\right|}$$

(3) $$W_{c}[i]=\frac{W_{c}[i]}{\sum_{i=0}^{L-1}W_{c}[i]}$$

where $c\in \left\{r,\;g,\;b\right\}$ is the channel index. We normalize $W_{c}$ into the range $\left(0,\;1\right]$ to obtain the relative importance for each grayscale level within each channel. To exclude zero denominator, 0 is not included. For pixel intensity, the closer the channel CDF is to the optimal CDF, the higher its relative importance score.

Once we have *W_C_*, we compute the weighted sum of each color pixel to get the grayscale output:

(4) $$Y\left[x,y\right]=\sum_{c\in \left\{r,g,b\right\}}\frac{W_{c}\left[I_{c}\left[x,y\right]\right]\cdot I_{c}\left[x,y\right]}{W\left[I_{c}\left[x,y\right]\right]}$$

where Y is the output grayscale image; $x\in \left[0,\;N-1\right]$ and $y\in \left[0,\;M-1\right]$ are the column and row index; M and N are the number of image rows and columns, respectively; $I_{c}$ is channel image c, $W\left[I_{c}[x,y]\right]=\sum_{c}W_{c}\left[I_{c}\left[x,y\right]\right]$ to ensure that the sum of the weights for a pixel color components equals 1. For the sample image in Figure 2a, the final gray scale image generated according to this method is given in Figure 2e. 

In the above equation, we can use a look-up-table (LUT) strategy ($W_{c}\left[I_{c}\left[x,y\right]\right]$) to obtain weights for pixel color components once the initial weights have been calculated. Also, our method works with the original RGB space directly, so no additional color space conversion is needed. The primary computational cost difference between the traditional methods and the proposed method is per-channel weight computation. For high-resolution images captured by modern devices, this may bring substantial additional computational cost, which may be a challenge for low-power CPUs or edge computing devices. A possible solution will be discussed in the next sub-section.

### 3.2. CDF Approximation

An important consideration in the design of the proposed method is that it should be as simple and fast as possible so that the computational cost will not be a concern when it is used in real applications. As we discuss above, the proposed method may bring substantial additional computational cost compared to the traditional method when the input image resolution is very high, as will commonly be the case with, e.g., high-resolution images captured by modern smartphones. So, we should consider this problem, especially for resource-constrained devices. One possible solution is to perform image sub-sampling. That is, we simply sub-sample the input image at a fixed sampling step s to approximate the true underlying CDFs. This approach works well due to inherent characteristics of natural images, whereby objects are somewhat continuous in the image.

**Figure 4:**
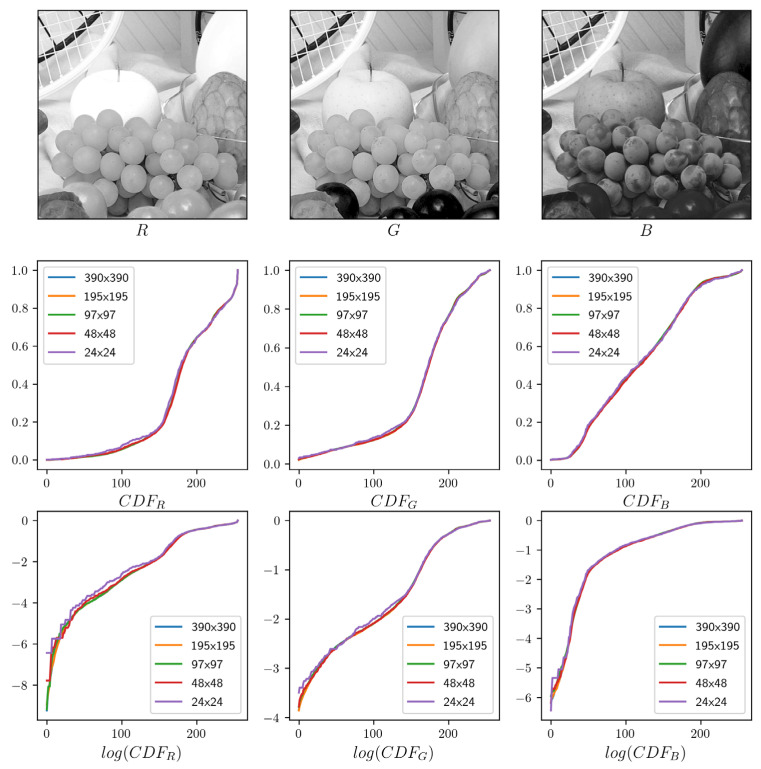
Effect of image sub-sampling on CDF estimation.

By way of an example, in Figure 4, we sub-sample the input image with step s∈[1, 2, 4, 8, 16] and show the effect of this on the CDF. The differences among the output CDFs at different sub-sampling steps are small, even in the logarithm space (the third row). Therefore, we expect that small differences will not have a significant impact on the conversion process.

## 4. Experiments

As we claim above, the proposed method can preserve more image contrast and details than the traditional methods. In this section, we compare the proposed method to several other ID algorithms with several objective metrics. We also compare the execution speed of our method with the Decolorize method of [29] as a benchmark; this algorithm is claimed to have linear computational cost as a function of image size (number of pixels). 

### 4.1. Performance Metrics

To compare performance, we require suitable objective metrics. Unfortunately, there are very few metrics specifically designed for this particular task. Reference image-based metrics that attempt to capture structural fidelity, like SSIM [39], are not suitable here because we do not have the ground-truth grayscale version of an input color image. Many of the well-known non-reference-image-based quality assessment methods, like BRISQUE [40], are not suitable here because they are designed for quality assessment of natural color images.

Based on the above discussion, we initially chose lower-level metrics such as the root mean square contrast difference (RMS) [41] to measure image contrast. RMS is appropriate for this application as it is a non-learning and non-reference-based objective metric for contrast measuring and is widely used in image quality measurement [42,43,44]. A larger RMS value means greater global image contrast. RMS is defined as:

(5) $$RMS=\sqrt{\frac{1}{MN}\sum_{x=0}^{N-1}\sum_{y=0}^{M-1}{\left(I\left[x,y\right]-\bar{I}\right)}^{2}}$$

where $I\left[x,y\right]$ is pixel intensity at location (x,y) $\bar{I}$ is mean intensity of the image, and *M*, *N* are the image dimensions. We used RMS to measure the contrast of the converted grayscale image of different algorithms.

We also extended the normalized root mean squared contrast difference (NRMS) to color images so that we can measure deviations between the converted grayscale image and the color channel images. This is an important metric to ensure that the final output does not have very different appearance from the input image:

(6) $$NRMS=\left(\sum_{c}\frac{\left\|I_{c}-Y\right\|}{I_{c}}\right)/3$$

where $\left\|\cdot \right\|$ denotes the Frobenius norm and Y is the final output image. For this metric, the smaller the better.

To measure image detail preservation, two new metrics were proposed. These two new measures are used to capture image details which are important for image analysis and computer vision tasks.

Gradient Recall Ratio (GRR): Gradient information is a very important component of image detail and is used in many popular computer vision algorithms, such as feature extraction for HoG [2] and Seam Carving [45]. So, we propose the *GRR* metric to measure how much gradient information has been retained in the grayscale output image.

For an input color and its corresponding grayscale image, we computed the gradient image for each channel image in the original RGB image and the corresponding grayscale image. Then, we evaluated the *GRR* as follows:

(7) $$GRR=\frac{\sum_{x,y}\sqrt{{Y_{x}\left[x,y\right]}^{2}+{Y_{y}\left[x,y\right]}^{2}}}{\sum_{c}\sum_{x,y}\sqrt{{I_{x}^{c}\left[x,y\right]}^{2}+{I_{y}^{c}\left[x,y\right]}^{2}}}$$

where $I_{x}^{c}$, $I_{y}^{c}$, $Y_{x}$, and $Y_{y}$ are channel and grayscale image derivatives along x and y. A higher score indicates better retention of gradient information. 

Edge Recall Ratio (ERR): Image edges are one of the fundamental sources of information for many computer vision tasks, for example line detection [7] and shape recognition [46]. This metric is used to measure how faithfully edge information is preserved in the image decolorization. We used the Canny operator [5] on each color image channel to obtain the whole edge mask:

(8) $$E_{rgb}=\left(E_{r}\vee E_{g}\vee E_{b}\right)$$

where $E_{rgb}$, $E_{r}$, $E_{g}$, and $E_{b}$ are edge masks of the original color image and the *R*, *G*, *B* color channels, respectively; ∨ is the logical OR operator. The edge mask values are 1 if a point is on an edge; otherwise, they are 0. 

We also ran the Canny operator on the corresponding grayscale image to obtain $E_{Y}$. Then, we defined the *ERR* as follows:

(9) $$ERR=\frac{\sum_{x=0}^{N-1}\sum_{y=0}^{M-1}\left(\left(E_{rgb}\left[x,y\right]⊕SE\right)\wedge E_{Y}\right)\left[x,y\right]}{\sum_{x=0}^{N-1}\sum_{y=0}^{M-1}E_{rgb}\left[x,y\right]}$$

where ∧ is logical AND operator, ⊕ is morphological dilation operator, and *SE* is a morphological structural element. The reason we used morphological dilation operator is because there may be some degree of edge shifting among color channels caused by chromatic aberration. This may be brought into the grayscale image during the conversion. By using dilation, shifting in the range $\left[-\left\lfloor \frac{r}{2}\right\rfloor ,\left\lfloor \frac{r}{2}\right\rfloor \right]$ can be tolerated, where r is the radius of structural element. In our experiment, we used a square structural element of size 5. For this metric, the higher the score, the more edge information has been preserved. Due to the dilation operation, this score may be larger than 1.

### 4.2. Results

**Figure 5:**
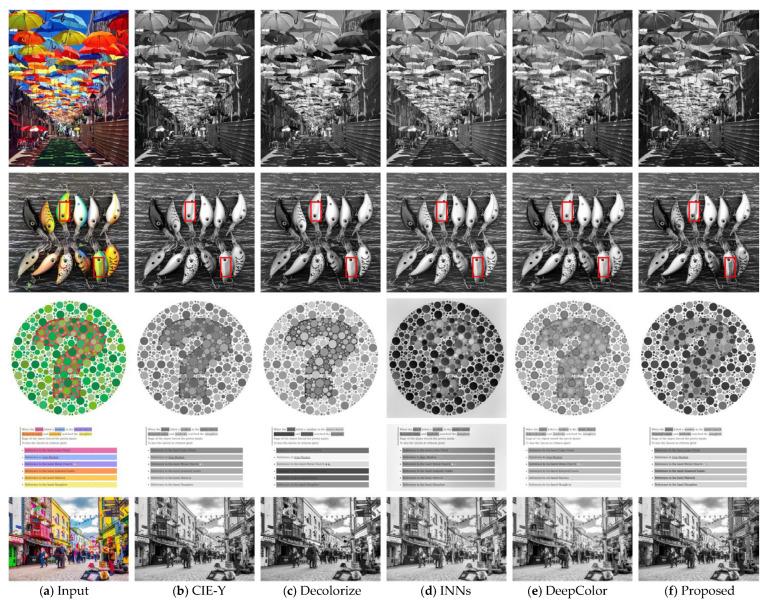
Some results from different ID algorithms.

We compared the proposed method to several classical algorithms and two modern deep learning-based algorithms from the literature. Figure 5 shows some example results. Based on visual inspection, the proposed method can retain more image details and image contrast than the traditional method (CIE-Y). In comparison with the other benchmark methods, our method consistently produces visually appealing output. For example, in the second row, many salient features and texture of surfaces are retained well in the grayscale image with the proposed algorithms.

To compare the algorithms objectively, we used the standard dataset built by Cadik [47] in our experiments. This dataset was designed specifically for the evaluation of ID algorithms and contains a carefully selected set of 25 images, including both natural and artificial images. Table 1 gives the *RMS* scores of each algorithm for each test image in the dataset presented in [47]. The best score for each test image (each row) is highlighted in bold text. There is some variation in that different algorithms perform better with different test images, but the proposed algorithm achieves the best overall score, which is much higher than the traditional CIE-Y method. This shows that the adaptive weights which are computed based on CDF can combine every color pixel effectively to produce a grayscale output with high image contrast. This improves visual appearance because the human vision system is more sensitive to image contrast than absolute luminance.

Table 2 presents the detailed NRMS scores. Our algorithm achieved the best overall performance along with two other methods [20,32]. This is because the proposed algorithm is still a linear combination of channel images, but different from traditional methods [20] in that different color pixels will have different weights to combine them. No low-level image information (e.g., image edges and textures) is used in the conversion process. As a result, the conversion process will not cause large deviation from the original input image. This shows that the proposed algorithm can retain the original illuminance appearance very well.

Table 3 captures the *GRR* scores of the different algorithms. Again, our algorithm achieves the best performance with this metric. This result coincides with the *RMS* score: high image contrast output helps to retain more image gradient information, which is vital information for many computer vision algorithms, as we mentioned before. These experimental results also show that different ID algorithms will cause different degrees of image detail information loss, which is important in many computer vision tasks. Table 4 presents the *ERR* scores. As expected, *ERR* scores are consistent with *GRR* scores because gradient information is key information for edge detection. We propose that this metric is not a simple duplicate of the GRR because sometimes, high image gradient information does not mean that an image contains lots of edges; for example, in an image with dark background which has many isolated white pixels. 

### 4.3. Speed Assessment

As noted above, the proposed method is nearly as fast as the traditional simple methods yet provides superior performance on average across all the metrics considered. In this sub-section, we ran a simple speed test for three methods, CIE-Y, Decolorize [29], and the proposed method, to illustrate this point. The experiment was simple: we executed the three algorithms with a high-resolution image (e.g., 4928 × 3280) hundreds of times. At each step, we down-sampled the input image by a factor of 2. We implemented the proposed method in Python and in C++ (single thread) without using any specific optimizations. Except the last column in Table 5, all were implemented in Python.

From Table 5, we can see that the computational load of all methods is approximately linear with image size (reflected in the down-sampling rate, denoted by “Scale” in the table): for each scale value, the execution time is reduced to approximately 1/4 of the previous value. For C++ implementation (without CDF approximation), our method can achieve 8 FPS with the original 15 MP image with mobile Intel i7 CPU without any optimization techniques applied. We believe that the execution speed of the proposed method can be boosted significantly by using, e.g., Single Instruction Multiple Data (SIMD) instructions such as Streaming SIMD Extensions (SSE) and Advanced Vector Extensions (AVX). There is also scope for performance boosting by implementing GPUs.

**Figure 6:**
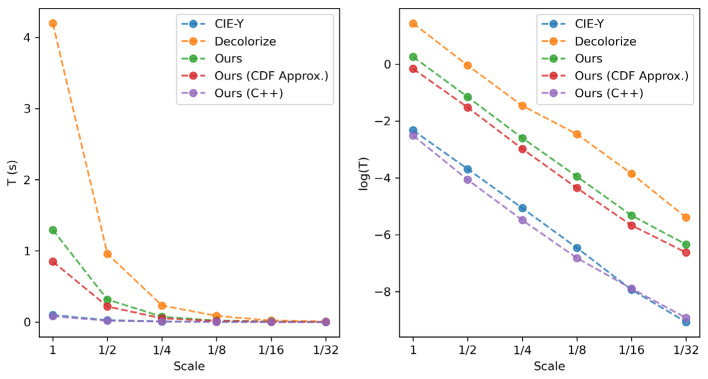
Runtime of the algorithms.

The logarithm of the execution time of each algorithm as a function of scale is plotted in Figure 6. Both linear and logarithmic plots are presented in order to both illustrate large differences in execution time at low-scale values as well as show the detail at higher-scale values. The unoptimized C++ implementation is slightly faster than the simple CIE-Y method, which is implemented in Python with NumPy [48]. NumPy is supported by the highly optimized Intel MKL library, which provides performance optimization. So, while it is difficult to directly compare implementations on different software platforms, the run-time figures in Table 5 and Figure 6 suggest that the computational cost of the proposed method can be comparable to the CIE-Y traditional method with appropriate optimization.

## 5. Conclusions

In this paper, we propose an efficient and effective image decolorization algorithm. It not only preserves image contrast and detail from the input color image, but also has nearly the same computational complexity as the simple CIE-Y method used as a benchmark, while providing a performance advantage in terms of structural (edge) information preservation. To evaluate its performance objectively, two new metrics are proposed. Experimental results show our method can achieve the best overall performance across all metrics. The proposed method has potential to be a good alternative to the traditional methods in terms of balancing the need to preserve image contrast and detail without significant additional computational cost.

While the proposed algorithm exhibits a number of advantages, there is scope for further investigation and improvement. For example, instead of using the CDF, alternative measures for image content may be used, as well as a more effective distance metric to compare the difference between the optimal measure and each channel image measure. Also, the impact of the image decolorization process on the performance of feature extraction or other computer vision algorithms (e.g., HoG [2], SIFT [3]) is of interest. These are areas for future research.

## Figures and Tables

**Table 1:** RMS scores (higher is better, the best score is in bold text).

Image Name	Smith [32]	CIE-Y [20]	Decolorize [29]	Color2Gray [22]	Neumann [24]	INNs [35]	DeepColor [36]	Proposed
butterfly	0.74	0.72	0.72	0.48	**0.77**	0.61	0.70	0.73
155_5572	0.40	0.40	0.42	0.45	0.53	0.40	0.39	**0.53**
dscn9952	0.53	0.49	0.44	0.54	0.74	0.47	0.45	**0.91**
im2-color	0.06	0.05	0.15	**0.29**	0.16	0.04	0.15	0.20
colorspastel	0.06	0.06	0.13	**0.35**	0.13	0.05	0.10	0.06
25_color	0.40	0.38	0.67	0.52	0.45	0.36	**0.84**	0.56
balls0_color	0.40	0.39	0.38	0.46	0.44	0.39	0.47	**0.59**
impatient	0.44	0.40	0.50	0.36	0.41	0.39	0.41	**0.53**
c8tz7768	0.30	0.29	**0.48**	**0.48**	0.33	0.28	0.28	0.29
sunrise312	0.18	0.16	**0.33**	0.26	0.20	0.15	0.19	0.31
monarch	0.42	0.37	0.38	0.38	0.39	0.36	0.35	**0.48**
serrano	0.49	0.47	0.51	0.46	**0.53**	0.41	0.46	0.48
girl	0.76	0.72	0.63	0.65	**0.78**	0.69	0.62	0.73
ski_tc8	0.66	0.62	0.60	0.43	**0.71**	0.56	0.62	0.70
watch	**0.60**	0.50	0.53	0.29	0.55	0.46	0.47	0.57
arctichare	0.21	0.21	**0.26**	0.22	0.22	0.19	0.24	0.23
ramp	0.03	0.03	0.19	**0.52**	0.16	0.02	0.06	0.08
text	**0.28**	0.25	0.27	0.21	0.26	0.19	0.27	0.26
colorwheel	0.08	0.03	0.33	**0.42**	0.22	0.04	0.30	**0.42**
tulips	0.60	0.53	**0.71**	0.46	0.57	0.53	0.66	0.64
fruits	0.28	0.28	0.35	0.26	0.31	0.25	0.28	**0.42**
kodim03	0.41	0.38	0.41	**0.42**	0.40	0.39	0.34	0.39
portrait	0.53	0.50	0.51	0.42	0.52	0.46	0.43	**0.54**
34445	0.63	0.58	0.61	0.37	0.62	0.49	0.58	**0.64**
tree_color	0.79	0.73	0.75	0.65	0.77	0.74	0.69	**0.80**
Overall average	0.41	0.38	0.45	0.41	0.45	0.36	0.41	**0.48**

**Table 2:** NRMS scores (lower is better, the best score is in bold text).

Image Name	Smith [32]	CIE-Y [20]	Decolorize [29]	Color2Gray [22]	Neumann [24]	INNs [35]	DeepColor [36]	Proposed
butterfly	0.18	0.17	0.17	0.32	0.19	0.34	0.17	**0.16**
155_5572	0.63	0.62	0.72	0.69	0.61	**0.58**	0.59	0.62
dscn9952	0.63	0.62	0.81	0.72	0.62	**0.61**	0.70	0.69
im2-color	**0.25**	**0.25**	0.28	0.43	0.33	**0.25**	**0.25**	**0.25**
colorspastel	**0.13**	**0.13**	0.15	0.36	0.21	**0.13**	0.15	**0.13**
25_color	0.35	0.37	**0.27**	0.32	0.33	0.37	0.32	0.28
balls0_color	0.42	0.42	0.56	0.43	0.40	0.43	**0.38**	0.41
impatient	0.39	0.39	0.47	0.46	0.38	0.39	0.39	**0.37**
c8tz7768	0.30	0.30	0.36	0.50	0.32	0.35	0.32	**0.29**
sunrise312	**0.18**	**0.18**	0.27	0.23	0.19	0.19	0.19	0.23
monarch	**0.30**	**0.30**	0.36	0.42	0.31	0.31	0.36	0.32
serrano	**0.34**	**0.34**	0.38	0.45	0.36	**0.34**	0.35	0.35
girl	**0.19**	**0.19**	0.25	0.22	**0.19**	**0.19**	0.22	**0.19**
ski_tc8	0.31	**0.30**	0.37	0.42	0.31	0.32	0.33	**0.30**
watch	**0.13**	**0.13**	0.16	0.32	0.14	0.21	0.25	**0.13**
arctichare	0.08	0.06	0.10	0.10	0.07	0.07	0.10	0.07
ramp	0.21	**0.20**	0.26	0.53	0.27	**0.20**	0.22	0.22
text	0.09	0.08	0.08	0.11	0.08	0.16	0.09	**0.07**
colorwheel	0.54	0.54	0.53	0.78	0.58	0.55	**0.50**	0.51
tulips	**0.31**	**0.31**	0.42	0.47	**0.31**	0.33	0.32	0.32
fruits	**0.24**	0.25	0.26	0.30	0.28	0.26	0.25	0.26
kodim03	0.31	0.31	0.33	0.38	0.32	0.30	0.35	**0.29**
portrait	**0.12**	**0.12**	0.14	0.19	0.13	0.17	0.16	**0.12**
34445	**0.13**	**0.13**	0.15	0.25	0.13	0.20	0.14	**0.13**
tree_color	0.07	0.07	0.07	0.15	0.07	0.09	0.11	**0.06**
Overall average	**0.27**	**0.27**	0.32	0.38	0.29	0.29	0.29	**0.27**

**Table 3:** GRR scores (higher is better, the best score is in bold text).

Image Name	Smith [32]	CIE-Y [20]	Decolorize [29]	Color2Gray [22]	Neumann [24]	INNs [35]	DeepColor [36]	Proposed
butterfly	**0.35**	0.32	0.33	0.23	0.32	0.19	**0.35**	0.33
155_5572	0.31	0.28	0.30	0.25	0.29	0.21	0.33	**0.45**
dscn9952	0.39	0.30	0.40	0.33	0.29	0.17	0.33	**0.57**
im2-color	0.12	0.03	0.20	**0.45**	0.30	0.10	0.29	0.31
colorspastel	0.23	0.21	0.38	**0.47**	0.33	0.19	0.33	0.22
25_color	0.26	0.24	**0.33**	0.28	0.27	0.21	0.32	0.31
balls0_color	0.34	0.3	0.31	0.29	0.35	0.26	0.34	**0.46**
impatient	0.31	0.27	0.32	0.27	0.29	0.23	0.31	**0.40**
c8tz7768	0.25	0.20	0.33	**0.40**	0.21	0.23	0.28	0.36
sunrise312	0.34	0.28	0.37	0.41	0.26	0.23	0.31	**0.46**
monarch	0.35	0.31	0.35	0.32	0.31	0.29	0.34	**0.39**
serrano	**0.35**	0.31	0.31	0.26	0.32	0.24	**0.35**	0.32
girl	**0.38**	0.32	0.35	0.31	0.33	0.27	0.34	**0.38**
ski_tc8	**0.37**	0.31	0.33	0.25	0.32	0.25	0.35	0.34
watch	**0.38**	0.33	0.36	0.24	0.35	0.27	0.35	0.35
arctichare	0.34	0.33	**0.37**	**0.37**	0.33	0.26	0.36	0.34
ramp	0.15	0.06	0.33	**0.53**	0.27	0.08	0.30	0.25
text	**0.36**	0.33	0.33	0.26	0.34	0.21	0.34	0.34
colorwheel	0.12	0.04	0.21	0.24	0.15	0.12	0.28	**0.30**
tulips	0.34	0.31	0.26	0.24	0.3	0.26	0.34	**0.37**
fruits	0.33	0.30	0.33	0.25	0.31	0.23	0.33	**0.41**
kodim03	**0.38**	0.33	0.32	0.36	0.33	0.26	0.36	**0.38**
portrait	**0.36**	0.32	0.35	0.31	0.34	0.26	0.34	0.34
34445	**0.36**	0.32	0.32	0.24	0.34	0.22	0.33	0.35
tree_color	0.36	0.31	0.32	0.34	0.33	0.24	0.31	0.33
Overall average	0.31	0.27	0.32	0.32	0.30	0.22	0.33	**0.36**

**Table 4:** ERR scores (higher is better, the best score is in bold text).

Image Name	Smith [32]	CIE-Y [20]	Decolorize [29]	Color2Gray [22]	Neumann [24]	INNs [35]	DeepColor [36]	Proposed
butterfly	0.83	0.82	0.81	0.79	0.83	0.78	0.83	**0.87**
155_5572	0.52	0.48	0.50	0.44	0.54	0.37	0.60	**0.75**
dscn9952	**0.67**	0.61	0.66	0.61	0.49	0.38	0.59	0.57
im2-color	0.50	0.00	0.81	0.78	0.86	0.00	**0.91**	0.89
colorspastel	0.57	0.58	0.76	0.63	0.58	0.50	**0.71**	0.61
25_color	0.97	0.97	0.97	0.97	0.96	0.99	0.97	**1.17**
balls0_color	0.61	0.59	0.60	0.59	0.65	0.55	0.61	**0.85**
impatient	0.39	0.35	0.45	0.39	0.38	0.29	0.40	**0.63**
c8tz7768	0.61	0.45	0.70	**0.79**	0.50	0.50	**0.79**	0.75
sunrise312	0.62	0.51	0.64	**0.74**	0.41	0.34	0.56	0.70
monarch	0.66	0.62	**0.67**	0.64	0.63	0.59	0.66	0.67
serrano	**0.76**	0.74	0.71	0.70	0.73	0.65	**0.76**	**0.76**
girl	**0.80**	0.65	0.77	0.63	0.60	0.43	0.68	0.76
ski_tc8	**0.73**	0.66	0.71	0.59	0.65	0.57	0.67	0.70
watch	0.85	0.82	**0.89**	0.79	0.84	0.76	0.84	0.86
arctichare	0.64	0.62	0.64	**0.68**	0.62	0.43	0.63	0.65
ramp	0.25	0.00	0.69	0.31	**0.80**	0.00	0.22	0.51
text	0.92	0.93	0.93	0.92	0.93	**0.96**	0.93	0.93
colorwheel	0.57	0.00	0.75	0.79	0.68	0.30	**0.86**	**0.86**
tulips	0.70	0.66	0.52	0.59	0.66	0.53	0.67	**0.73**
fruits	0.56	0.53	0.59	0.42	0.53	0.36	0.58	**0.73**
kodim03	0.77	0.72	0.69	0.73	0.68	0.53	0.73	**0.78**
portrait	**0.73**	0.70	**0.73**	0.72	**0.73**	0.63	0.72	0.72
34445	**0.77**	0.76	0.76	0.75	**0.77**	0.71	0.76	0.76
tree_color	0.61	0.52	0.59	**0.62**	0.58	0.38	0.53	0.57
Overall average	0.66	0.57	0.70	0.66	0.67	0.50	0.69	**0.75**

**Table 5:** Runtime comparison (seconds).

Scale	CIE-Y	Decolorize	Proposed	Proposed (CDF Approx.)	Proposed (C++)
1	0.1447	8.5286	1.1775	0.7790	0.1204
1/2	0.0409	1.5657	0.2969	0.1982	0.0280
1/4	0.0103	0.3394	0.0828	0.0510	0.0068
1/8	0.0022	0.0698	0.0204	0.0121	0.0015
1/16	0.0009	0.1492	0.0052	0.0030	0.0009
1/32	0.0007	0.0043	0.0019	0.0012	0.0007

## Data Availability

Data is contained within the article.
